# Ultrasound-assisted aqueous extract of *Campanula macrostachya* enhances fibroblast repair via antioxidant and cytokine-modulatory mechanisms

**DOI:** 10.1007/s00709-026-02192-z

**Published:** 2026-04-01

**Authors:** Cengiz Sarikurkcu, Fatma Ozlem Kargin Solmaz, Nilay Isitez, Sevim Feyza Erdoğmuş

**Affiliations:** Faculty of Pharmacy, Afyonkarahisar Health Sciences University, Afyonkarahisar, Türkiye

**Keywords:** *Campanula macrostachya*, Wound healing, Antioxidant activity, Cytokine modulation, Fibroblast migration

## Abstract

Plants are valuable sources of bioactive metabolites that influence cellular physiology and tissue repair. This study investigates the phytochemical composition and cellular wound-healing potential of the aqueous extract of *Campanula macrostachya* Boiss. (Campanulaceae), an endemic Anatolian species traditionally used for treating skin injuries. The extract was obtained via ultrasound-assisted extraction and characterized by LC–MS, revealing chlorogenic and caffeic acids as the dominant phenolics. Antioxidant properties were evaluated using DPPH, ABTS, FRAP, CUPRAC, phosphomolybdenum, and ferrous ion chelating assays, while enzyme inhibition screening included acetylcholinesterase, butyrylcholinesterase, tyrosinase, α-amylase, and α-glucosidase inhibitory activities. In human dermal fibroblast (HDFa) cultures, the extract exhibited no cytotoxicity up to 62.5 µg/mL, significantly enhanced cell viability, and promoted 98% wound closure within 48 h in scratch assays. Moreover, TNF-α and IL-1β levels were downregulated, suggesting an anti-inflammatory contribution to tissue repair. These findings provide mechanistic insights into the cellular basis of *C. macrostachya*’s ethnopharmacological use and support its potential as a source of phenolic compounds that modulate redox balance and cytokine activity. The study highlights the relevance of plant-derived antioxidants in regulating fibroblast behavior and suggests their potential for future development of natural agents for skin-related applications, based on in vitro findings.

**Supplementary Information:**

The online version contains supplementary material available at 10.1007/s00709-026-02192-z.

## Introduction

Wound healing is a dynamic and highly coordinated biological process that restores tissue integrity and functionality following injury. It involves a sequence of overlapping events—hemostasis, inflammation, proliferation, and remodeling—each governed by complex cellular and molecular interactions (Gurtner et al. 2008; Martin and Leibovich 2005; Schäfer and Werner 2007). Several intrinsic and extrinsic factors, including aging, oxidative stress, metabolic disorders, and microbial infection, can interfere with these mechanisms, resulting in delayed or chronic wounds. To overcome such challenges, increasing attention has been directed toward plant-derived bioactive compounds as natural and multi-targeted therapeutic agents capable of modulating different stages of the wound healing cascade. Phytochemicals such as flavonoids, terpenoids, tannins, and phenolic acids are known to promote tissue regeneration through antioxidant, antimicrobial, and anti-inflammatory properties, as well as by stimulating collagen synthesis and angiogenesis (Fronza et al. 2009; Ghosh and Gaba 2013).

Within this context, species belonging to the genus *Campanula* L. (Campanulaceae) have attracted interest for their ethnomedicinal and pharmacological significance. The genus, commonly known as bellflower, comprises approximately 420 species distributed mainly across temperate regions of the Northern Hemisphere, with centers of diversity in the Mediterranean and Caucasus. Turkey represents a major biodiversity hotspot, harboring around 120 *Campanula* taxa, nearly half of which are endemic (Erdogan et al. 2025; Yilmaz et al. 2023). Several species are traditionally used in folk medicine as wound-healing agents, diuretics, antidiabetics, and digestive remedies (Erdogan et al. 2025; Marah et al. 2024). In addition to their medicinal relevance, some taxa such as *C. pelviformis* are consumed as edible greens within the Mediterranean diet, providing essential nutrients and phytochemicals with antioxidant and anti-inflammatory activities (Tsiftsoglou et al. 2023).

Phytochemical investigations have revealed that *Campanula* species are rich in flavonoids, phenolic acids, triterpenes, coumarins, and polyacetylenes, compounds largely responsible for their biological effects. Studies on Turkish *Campanula* species, including *C. peregrina*, *C. cymbalaria*, *C. davisii*, and *C. stricta var. libanotica*, have demonstrated high total phenolic and flavonoid contents associated with potent antioxidant activity as evidenced by DPPH, ABTS, FRAP, and CUPRAC assays (Erdogan et al. 2025). The presence of chlorogenic, caffeic, and p-coumaric acids, along with flavonoids such as rutin, quercetin, and kaempferol, has been linked to these antioxidant effects. Similarly, endemic species like *C. baskilensis* exhibit high concentrations of quinic, protocatechuic, vanillic, and chlorogenic acids, as well as flavonoid glycosides such as hesperidin and nicotiflorin, which contribute to significant enzyme inhibitory and antioxidant properties (Marah et al. 2024; Yilmaz et al. 2023). These findings collectively support the ethnopharmacological use of *Campanula* species and underscore their potential as sources of therapeutically valuable phytochemicals.

Among these taxa, *Campanula macrostachya* Boiss., an endemic species distributed in southwestern Anatolia, has recently garnered scientific interest. Previous phytochemical studies identified it as a rich source of phenolic acids—predominantly chlorogenic, caffeic, protocatechuic, and syringic acids—contributing to its notable antioxidant and enzyme inhibitory capacity (Sarikurkcu et al. 2021). Given that oxidative stress plays a crucial role in delayed wound healing and chronic inflammation, phenolic-rich extracts from *C. macrostachya* may offer significant therapeutic potential for promoting tissue repair. Impaired wound healing is frequently associated with metabolic disorders such as diabetes mellitus, where persistent hyperglycemia exacerbates oxidative stress, sustains pro-inflammatory signaling, and disrupts fibroblast migration and extracellular matrix remodeling (Brem and Tomic-Canic 2007; Falanga 2005). In this context, inhibition of carbohydrate-hydrolyzing enzymes, including α-amylase and α-glucosidase, represents an established strategy to attenuate postprandial hyperglycemia and thereby reduce glucose-induced oxidative and inflammatory damage at the cellular level (Apostolidis and Lee 2010; Tundis et al. 2010). Plant-derived phenolic compounds exhibiting α-amylase and α-glucosidase inhibitory activities have been reported to indirectly support wound healing by improving redox balance and modulating inflammatory pathways that are critical for fibroblast function and tissue repair (Herman and Herman 2023). Therefore, the evaluation of these enzyme inhibitory activities in the present study was included to provide complementary mechanistic insight into the potential relevance of *Campanula macrostachya* under conditions associated with impaired wound healing.

Accordingly, the present study aims to evaluate, for the first time, the wound healing potential of the aqueous extract of *C. macrostachya* obtained via ultrasound-assisted extraction. The study investigates its phytochemical composition, antioxidant capacity, cytocompatibility, and in vitro wound healing effects on human dermal fibroblasts (HDFa). By correlating the bioactive phenolic profile of the extract with cellular responses, this research provides mechanistic evidence supporting the traditional use of C. macrostachya and highlights its potential for future dermatological and tissue regeneration applications, based on in vitro findings.

## Materials and methods

### Plant material

The aerial parts of *Campanula macrostachya* were collected from the roadside along the Barla–Senirkent highway in Isparta Province, Turkey, during the flowering stage on 29 June 2025 (38° 05′ 13″ N, 30° 48′ 54″ E; 950–1000 m a.s.l.). The plant material was taxonomically re-identified by Dr. Olcay Ceylan, Department of Biology, Muğla Sıtkı Koçman University, and voucher specimens were deposited in the herbarium of the same institution. The aerial parts were dried under shade in a well-ventilated environment with low humidity and without direct exposure to sunlight. The dried material was subsequently ground into a fine powder using a laboratory blender.

### Preparation of the aqueous extract

Ultrasound-assisted extraction is a widely applied technique for the efficient recovery of phenolic compounds from plant matrices, as acoustic cavitation enhances cell wall disruption, facilitates solvent penetration, and improves mass transfer. The use of water as an extraction solvent is particularly suitable for the selective extraction of polar phenolics while ensuring low toxicity and environmental sustainability. Previous studies have demonstrated that ultrasound-assisted aqueous extraction provides higher phenolic yields and antioxidant activity compared to conventional extraction methods, especially for phenolic acid–rich plant materials (Azmir et al. 2013; Chemat et al. 2017; Sarikurkcu et al. 2021). Accordingly, an ultrasound-assisted extraction (UAE) technique was employed to obtain the aqueous extract, following the method described by Latiff et al. (2021) with slight modifications. Briefly, a sample-to-solvent ratio of 1:20 (w/v) was applied, and the extraction was carried out in a sonication bath at 30 °C for 1 h. After extraction, the mixture was filtered, frozen at − 18 °C, and subsequently lyophilized using a laboratory freeze-dryer. The extraction yield was calculated as 23.70%. The resulting lyophilized extract was stored at 4 °C until further analysis.

### Determination of chemical composition

The total flavonoid content (TFC) was determined by the aluminum chloride colorimetric method, and the total phenolic content (TPC) was quantified using the Folin–Ciocalteu reagent. Results were expressed as rutin equivalents (REs) and gallic acid equivalents (GAEs), respectively, according to the procedures described by Sarikurkcu et al. (2013). The detailed phytochemical composition was analyzed using a validated chromatographic method as reported by Cittan and Çelik (2018). Additional methodological parameters are provided in the Supplementary Material.

### Biological activity

The biological activity of the aqueous extract was evaluated using a comprehensive set of in vitro assays. Antioxidant activity was assessed by DPPH and ABTS radical scavenging assays, ferric reducing antioxidant power (FRAP), cupric ion reducing antioxidant capacity (CUPRAC), phosphomolybdenum total antioxidant capacity (Kocak et al. 2016), and ferrous ion chelating assays, following previously established protocols (Apak et al. 2006; Kocak et al. 2016; Sarikurkcu et al. 2020; Tepe et al. 2011).

Enzyme inhibitory activities were determined against acetylcholinesterase and butyrylcholinesterase using Ellman-based colorimetric methods, tyrosinase using the L-DOPA oxidation assay, and α-amylase and α-glucosidase using carbohydrate-hydrolyzing enzyme inhibition assays, as described in validated literature methods (Ozer et al. 2018; Sarikurkcu et al. 2020). Full experimental details for each assay are provided in the Supplementary Material.

### Cell culture and in vitro cytotoxicity assay

Human dermal fibroblast adult (HDFa) cells (ATCC, PCS-201-012) were cultured in Dulbecco’s Modified Eagle Medium (DMEM) supplemented with 10% fetal bovine serum (FBS) and 1% penicillin-streptomycin solution (Sigma-Aldrich, Germany). The cytotoxic potential of *C. macrostachya* extract was evaluated using the methyl thiazolyl tetrazolium (MTT) reduction assay, as previously described by Kumar et al. (2018). HDFa cells were seeded in 96-well plates at a density of 2 × 10⁴ cells per well and allowed to adhere until reaching 70–80% confluence. Subsequently, the cells were treated with various concentrations of the plant extract (1.9–1000 µg/mL), which was prepared by dissolving the extract in culture medium, and incubated for 24 h at 37 °C in a humidified atmosphere containing 5% CO₂.Following the incubation period, MTT reagent was added to each well, and the plates were further incubated at 37 °C for 2 h. After incubation, the supernatant was removed, and dimethyl sulfoxide (DMSO) was added to dissolve the resulting formazan crystals. The plates were gently agitated at low speed for 5 min at room temperature to ensure complete solubilization. Absorbance was measured at 570 nm using a microplate reader (Multiscan Sky, Thermo Fisher Scientific), and cell viability was expressed as a percentage relative to the untreated control group, which was considered 100% viable. To exclude potential assay interference, a cell-free control containing the extract and MTT reagent without cells was included, and no detectable absorbance was observed at 570 nm.The percentage of cell viability was calculated according to Eq. 1.

1 $$\:Cell\;viability\;(\%)=\begin{bmatrix}1&-\;\left(\frac{A_{570}of\;the\;test}{A_{570}of\;control}\right)\end{bmatrix}\;\times100$$

### Wound healing assay

The wound healing activity of *Campanula macrostachya* extract was evaluated using the CytoSelect™ 24-well wound healing assay (Cell Biolabs, San Diego, CA, USA) in HDFa cells. The concentration of 62.5 µg/mL, identified as the highest non-cytotoxic dose that promoted cell viability, was selected as the treatment concentration. The extract was prepared by dissolving it in the culture medium, and untreated cells were used as the control group. Sterile forceps were used to position the inserts designed to create wound areas into the wells, ensuring firm contact with the bottom surface and alignment of all wound fields in the same direction. A cell suspension containing 1.0 × 10⁶ cells/mL in DMEM supplemented with 10% (v/v) FBS was prepared in accordance with the CytoSelect™ kit protocol. Aliquots of 500 µL of this suspension were added to each well and incubated for 24 h to allow the formation of a uniform monolayer. Subsequently, the inserts were gently removed without disturbing the wound edges. The culture medium was carefully aspirated, and the wells were washed 4–6 times with serum-free medium to remove debris and non-adherent cells. Following washing, fresh medium containing 10% FBS was added, and the plant extract was applied to the corresponding wells (Fox et al. 2017). The cells were then incubated at 37 °C in a humidified atmosphere with 5% CO₂. Images of wound closure were captured at 0, 24, and 48 h using an inverted light microscope (Nikon Eclipse TS100) equipped with a Nikon digital camera at 10× magnification. The wound areas (diameter in µm) were quantified using ImageJ software, and the percentage of wound closure was calculated according to Eq. 2. Wound area measurements were performed using ImageJ software in triplicate (*n* = 3). For each experimental group, three independent wound images were analyzed, and the percentage of wound closure was calculated by normalizing the remaining wound area to the initial wound area at 0 h.

  

2 $$\begin{aligned}&Wound\;Closure\;(\%)=\lbrack\begin{array}{c}Migrated\;Cell\;Surface\;Area/\end{array}\\&Total\;Surface\;Area)\rbrack\;\times100\end{aligned}$$

### Preparation of the cell lysate

HDFa cells were maintained in DMEM supplemented with 10% FBS and 1% penicillin–streptomycin at 37 °C in a humidified atmosphere containing 5% CO₂ until they reached 70–80% confluence (approximately 24 h). The plant extract concentration of 62.5 µg/mL, determined as the highest non-cytotoxic dose that enhanced cell viability, was selected as the treatment concentration. The plant extract was prepared by dissolving it in the culture medium, while untreated cells served as the control group. The extract was added at a maximum volume corresponding to 1% of the total medium in the flask and incubated at 37 °C in a humidified atmosphere containing 5% CO₂ for 24 h. After the incubation period, the cells were detached from the flask surface by trypsinization, collected in Falcon tubes, and neutralized with complete culture medium. The cell suspensions were then centrifuged and washed twice with culture medium to remove residual trypsin. The resulting cell pellets were subsequently washed twice with ice-cold phosphate-buffered saline (PBS) and detached by gentle scraping. The harvested cells were lysed in lysis buffer containing 100 mM NaH₂PO₄, 1% Triton X-100, 1 M HEPES, and 1% protease inhibitor cocktail. The lysates were then centrifuged at 13,000 × g for 40 min at 4 °C, and the supernatants were collected for further analysis (Hazman et al. 2021).

### Effect of the plant extract on cytokine production

The total protein concentrations of the cell lysates were determined using the Bradford Protein Assay Kit (Quick Start, BIO-RAD). The total protein levels of the samples were used to normalise the cytokine data measured by the ELISA method. Therefore, the data obtained from the analysis of inflammatory cytokines tumor necrosis factor-α (TNF-α; E0082Hu, BT Lab, China), and interleukin 1 Beta (IL-1β; E0143Hu, BT Lab, China) for each sample were divided by the total protein level of each sample to obtain raw data. The normalised cytokine levels were expressed as ng/mg protein or pg/mg protein (Hazman et al. 2021).

### Statistical analysis

All experimental data are expressed as mean ± standard deviation (SD) of three independent biological replicates (*n* = 3), each analyzed in technical triplicate. Statistical analyses were performed using one-way analysis of variance (ANOVA) followed by Tukey’s post hoc test for multiple group comparisons, and Student’s t-test was used for comparisons between two groups. A significance level of *p* < 0.05 was considered statistically significant. All statistical analyses were conducted using SPSS software (version 22.0).

## Results

### Extraction yield and total phenolic and flavonoid contents

The extraction yield of the *C. macrostachya* aerial parts obtained by ultrasound-assisted aqueous extraction was 23.70%, indicating efficient recovery of polar phytochemicals. The total phenolic content of the extract was 46.76 mg gallic acid equivalents (GAEs)/g extract, while the total flavonoid content was 12.22 mg rutin equivalents (REs)/g extract (Table 1). The high recovery of phenolic acids observed in this study supports the suitability of ultrasound-assisted aqueous extraction for isolating polar bioactive compounds from *Campanula* species, in agreement with previous reports (Chemat et al. 2017; Sarikurkcu et al. 2021).

**Table 1:** Total phenolic and flavonoid contents of the aqueous extract from *C. macrostachya*

Assays	Aqueous extract
Total flavonoids (mg REs/g extract)	12.22 ± 0.31
Total phenolics (mg GAEs/g extract)	46.76 ± 0.49

Values are expressed as mean ± SD (*n* = 3). REs and GAEs rutin and gallic acid equivalents

### Phenolic composition of the aqueous extract

The phenolic profile of the aqueous extract was characterized by LC–MS analysis, through which 30 compounds were screened and 15 were identified (Table 2). Chlorogenic acid (2009 µg/g extract) and caffeic acid (1129 µg/g extract) were the predominant compounds, followed by 4-hydroxybenzoic acid (321 µg/g extract). Other phenolic acids such as 3-hydroxybenzoic, syringic, protocatechuic, and *p*-coumaric acids were also present in notable quantities. Among flavonoids, quercetin (28.4 µg/g) and kaempferol (24.1 µg/g) were the most abundant, whereas several compounds such as catechin, epicatechin, apigenin, and luteolin derivatives were not detected.

**Table 2:** Concentration of selected phenolic compounds in the aqueous extract from *C. macrostachya*

No	Compounds	Concentration (µg/g extract)
1	Chlorogenic acid	2009 ± 19
2	Caffeic acid	1129 ± 5
3	4-Hydroxybenzoic acid	321 ± 5
4	3-Hydroxybenzoic acid	296 ± 4
5	Syringic acid	137 ± 2
6	Protocatechuic acid	121 ± 2
7	p-Coumaric acid	114 ± 1
8	2,5-Dihydroxybenzoic acid	114 ± 1
9	Ferulic acid	71.5 ± 0.6
10	Pyrocatechol	68.5 ± 0.8
11	Pinoresinol	68.4 ± 2.4
12	Gallic acid	53.0 ± 0.2
13	Quercetin	28.4 ± 0.1
14	Kaempferol	24.1 ± 3.1
15	Hyperoside	3.02 ± 0.02
16	(-)-Epicatechin	nd
17	(+)-Catechin	nd
18	2-Hydroxycinnamic acid	nd
19	3,4-Dihydroxyphenylacetic acid	nd
20	Apigenin	nd
21	Apigenin 7-glucoside	nd
22	Eriodictyol	nd
23	Hesperidin	nd
24	Luteolin	nd
25	Luteolin 7-glucoside	nd
26	Rosmarinic acid	nd
27	Sinapic acid	nd
28	Taxifolin	nd
29	Vanillin	nd
30	Verbascoside	nd

Values are expressed as mean ± SD (*n* = 3). *nd* not detected

### Antioxidant activity

The antioxidant activity of the *C. macrostachya* aqueous extract was comprehensively evaluated using multiple in vitro assays that reflect distinct antioxidant mechanisms, including electron transfer, radical scavenging, and metal chelation (Table 3). The results were expressed both as half-maximal effective or inhibitory concentration (EC₅₀/IC₅₀, mg/mL) and in terms of the corresponding standard equivalents (Trolox or EDTA equivalents, mg TEs or mg EDTAEs per g extract).

**Table 3:** Antioxidant activity of the aqueous extract from *C. macrostachya*

Assays	Aqueous extract	Trolox	EDTA
Phosphomolybdenum (EC_50_: mg/ml)	0.80 ± 0.01^*b*^	0.54 ± 0.02^*a*^	-
CUPRAC reducing power (EC_50_: mg/ml)	0.86 ± 0.01^*b*^	0.14 ± 0.01^*a*^	-
FRAP reducing power (EC_50_: mg/ml)	0.33 ± 0.004^*b*^	0.042 ± 0.002^*a*^	-
DPPH radical scavenging (IC_50_: mg/ml)	1.43 ± 0.05^*b*^	0.14 ± 0.02^*a*^	-
ABTS cation radical scavenging (IC_50_: mg/ml)	0.58 ± 0.01^*b*^	0.088 ± 0.004^*a*^	-
Ferrous ion chelating (IC_50_: mg/ml)	2.66 ± 0.01^*b*^		0.019 ± 0.001^*a*^
Phosphomolybdenum (mg TEs/ g extract)	665.75 ± 7.30		
CUPRAC reducing power (mg TEs/ g extract)	167.27 ± 2.32		
FRAP reducing power (mg TEs/ g extract)	123.90 ± 1.64		
DPPH radical scavenging (mg TEs/ g extract)	91.95 ± 3.05		
ABTS cation radical scavenging (mg TEs/ g extract)	146.90 ± 1.79		
Ferrous ion chelating (mg EDTAEs/ g extract)	7.10 ± 0.03		

Values are expressed as mean ± SD (*n* = 3), and mean values with different superscript letters indicate statistically significant differences between groups in the same row, as determined by Student’s t-test (*p* < 0.05). TEs and EDTAEs, trolox and ethylenediaminetetraacetic acid (disodium salt) equivalents, respectively

The extract demonstrated a strong total antioxidant capacity in the phosphomolybdenum assay, with an EC₅₀ value of 0.80 mg/mL, which was significantly weaker than that of the reference antioxidant Trolox (EC₅₀ = 0.54 mg/mL), as indicated by different superscript letters in Table 3 (*p* < 0.05). When expressed as Trolox equivalents, the total antioxidant capacity of the extract reached 665.75 mg TEs/g extract. Similarly, in the CUPRAC and FRAP assays, which evaluate reducing power, the extract exhibited EC₅₀ values of 0.86 mg/mL and 0.33 mg/mL, corresponding to 167.27 mg TEs/g and 123.90 mg TEs/g extract, respectively. Compared with Trolox, the reducing capacities observed in these assays were significantly lower, yet they confirm a substantial electron-donating potential of the extract (Table 3, *p* < 0.05).

Free radical–scavenging capacity was evaluated using DPPH and ABTS assays, in which the extract exhibited IC₅₀ values of 1.43 mg/mL and 0.58 mg/mL, respectively. The corresponding Trolox equivalent antioxidant capacities were 91.95 mg TEs/g for DPPH and 146.90 mg TEs/g extract for ABTS. When compared with the reference antioxidant Trolox (IC₅₀ = 0.14 mg/mL for DPPH and 0.088 mg/mL for ABTS), the radical-scavenging activities of the extract were significantly lower, as indicated by different superscript letters in Table 3 (*p* < 0.05). Nevertheless, the extract displayed a measurable radical-scavenging potential, which may be attributed to the presence of redox-active phenolic acids such as chlorogenic (2009 µg/g) and caffeic (1129 µg/g) acids identified in Table 2.

In the ferrous ion–chelating assay, the extract exhibited a moderate chelating capacity, with an IC₅₀ value of 2.66 mg/mL, which was significantly weaker than that of the reference chelator EDTA (IC₅₀ = 0.019 mg/mL), as indicated by different superscript letters in Table 3 (*p* < 0.05). This activity corresponded to 7.10 mg EDTA equivalents (EDTAEs)/g extract. Taken together, these findings indicate that the aqueous extract of *C. macrostachya* exerts its antioxidant activity through multiple mechanisms—primarily electron donation and free radical scavenging—while displaying comparatively lower metal-chelating efficiency than EDTA.

### Enzyme ınhibition activity

The enzyme inhibition potential of the *C. macrostachya* aqueous extract was investigated against a series of clinically relevant enzymes, including acetylcholinesterase (AChE), butyrylcholinesterase (BChE), tyrosinase, α-amylase, and α-glucosidase (Table 4). The activities were quantified both as IC₅₀ values (mg/mL) and in terms of equivalent inhibitory capacities relative to specific reference compounds—galanthamine (GALAEs), kojic acid (KAEs), and acarbose (ACEs).

**Table 4:** Enzyme inhibition activity of the aqueous extract from *C. macrostachya*

Assays	Aqueous extract	Galanthamine	Kojic acid	Acarbose
Acetylcholinesterase inhibition (IC_50_: mg/ml)	5.79 ± 0.06^*b*^	0.0027 ± 0.0003^*a*^	-	-
Butyrylcholinesterase inhibition (IC_50_: mg/ml)	1.98 ± 0.02^*b*^	0.0033 ± 0.0003^*a*^	-	-
Tyrosinase Inhibition (IC_50_: mg/ml)	2.01 ± 0.01^*b*^	-	0.076 ± 0.005^*a*^	-
α-Amylase inhibition (IC_50_: mg/ml)	26.00 ± 0.75^*b*^	-	-	0.94 ± 0.02^*a*^
α-Glucosidase inhibition (IC_50_: mg/ml)	1.57 ± 0.004^*b*^	-	-	1.24 ± 0.05^*a*^
Acetylcholinesterase inhibition (mg GALAEs/g extract)	0.49 ± 0.01			
Butyrylcholinesterase inhibition (mg GALAEs/g extract)	1.57 ± 0.02			
Tyrosinase Inhibition (mg KAEs/g extract)	37.24 ± 0.17			
α-Amylase inhibition (mg ACEs/g extract)	35.55 ± 1.02			
α-Glucosidase inhibition (mg ACEs/g extract)	797.97 ± 2.16			

Values are expressed as mean ± SD (*n* = 3), and mean values with different superscript letters indicate statistically significant differences between groups in the same row, as determined by Student’s t-test (*p* < 0.05). ACEs, GALAEs and KAEs mean acarbose, galanthamine and kojic acid equivalents, respectively

The extract showed mild cholinesterase inhibition, with IC₅₀ values of 5.79 mg/mL for AChE and 1.98 mg/mL for BChE, corresponding to 0.49 mg GALAEs/g and 1.57 mg GALAEs/g extract, respectively. These inhibitory effects were significantly weaker than those of the reference compound galanthamine, as indicated by different superscript letters in Table 4 (*p* < 0.05), yet they confirm a measurable cholinesterase inhibitory activity.

Tyrosinase inhibition by the extract was moderate, with an IC₅₀ value of 2.01 mg/mL and an equivalent activity of 37.24 mg KAEs/g extract. When compared with the reference inhibitor kojic acid (IC₅₀ = 0.076 mg/mL), the inhibitory effect of the extract was significantly weaker, as indicated by different superscript letters in Table 4 (*p* < 0.05), indicating a mild tyrosinase inhibitory activity.

Regarding carbohydrate-hydrolyzing enzymes, the extract demonstrated pronounced α-glucosidase inhibition (IC₅₀ = 1.57 mg/mL; 797.97 mg ACEs/g extract), which was markedly stronger than its α-amylase inhibition (IC₅₀ = 26.00 mg/mL; 35.55 mg ACEs/g extract). When compared with the reference inhibitor acarbose, which exhibited IC₅₀ values of 0.94 mg/mL for α-amylase and 1.24 mg/mL for α-glucosidase, the inhibitory effects of the extract on both enzymes were significantly different, as indicated by different superscript letters in Table 4 (*p* < 0.05). These data indicate that *C. macrostachya* selectively inhibits α-glucosidase more efficiently than α-amylase, suggesting its potential utility in attenuating postprandial hyperglycemia through delayed glucose absorption.

### In vitro cytotoxicity assay

**Fig. 1:**
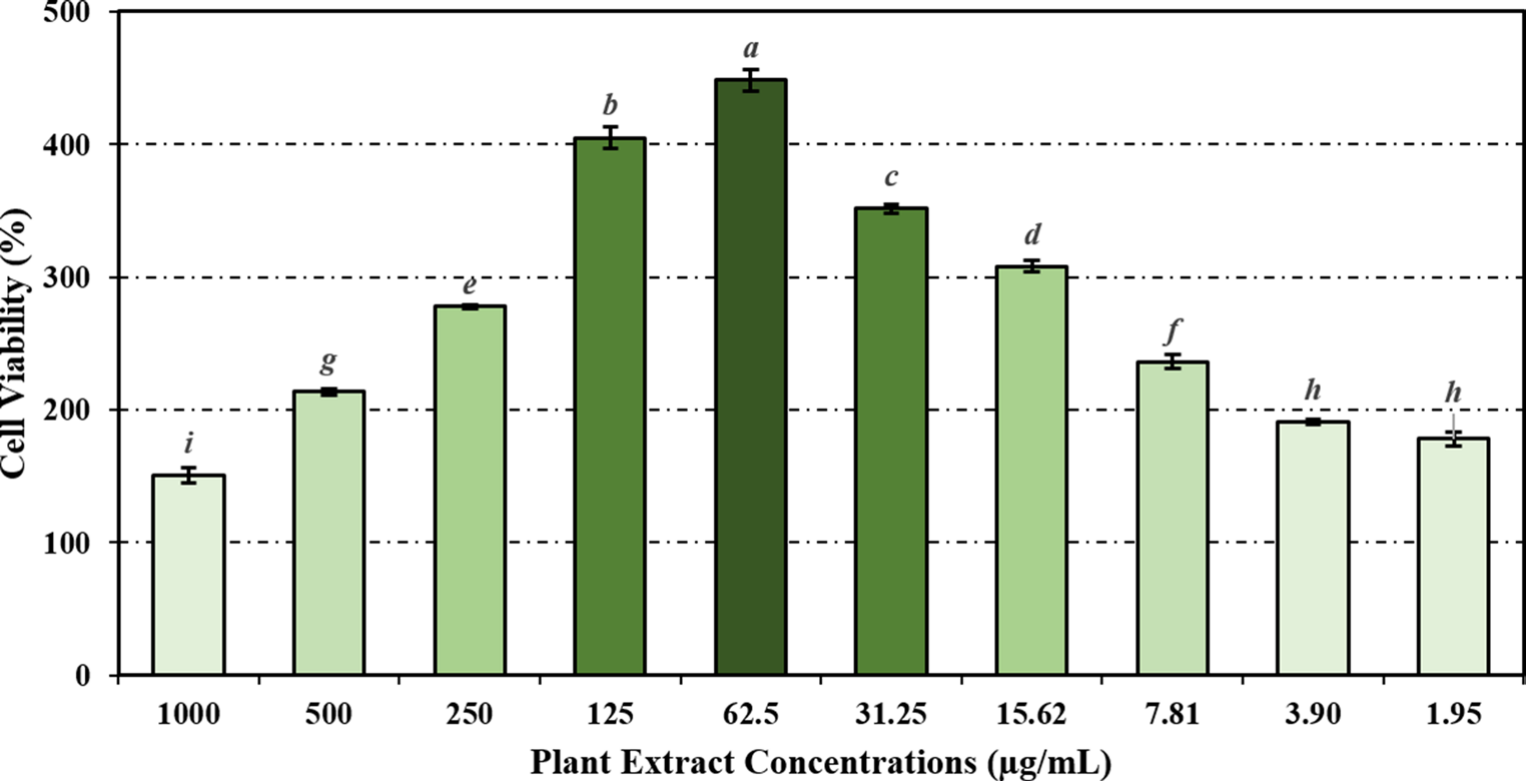
In vitro effect of *C. macrostachya* extract on HDFa cell viability at different concentrations. Values are expressed as mean ± SD (*n* = 3), and different superscript letters above the bars indicate statistically significant differences between concentrations, as determined by one-way ANOVA followed by Tukey’s post hoc test (*p* < 0.05)

The cytotoxic effect of the plant extract on HDFa cells was evaluated using the MTT assay, and the results are presented in Fig. 1. The concentration of 62.5 µg/mL was identified as the highest non-cytotoxic dose, which significantly enhanced cell viability (448.25 ± 8.04%) compared to the control group (*p* < 0.05). This increase reflects enhanced cellular metabolic activity rather than a direct increase in cell number. No direct chemical reduction of MTT by the extract was observed in cell-free controls.

### Wound healing assay

**Fig. 2:**
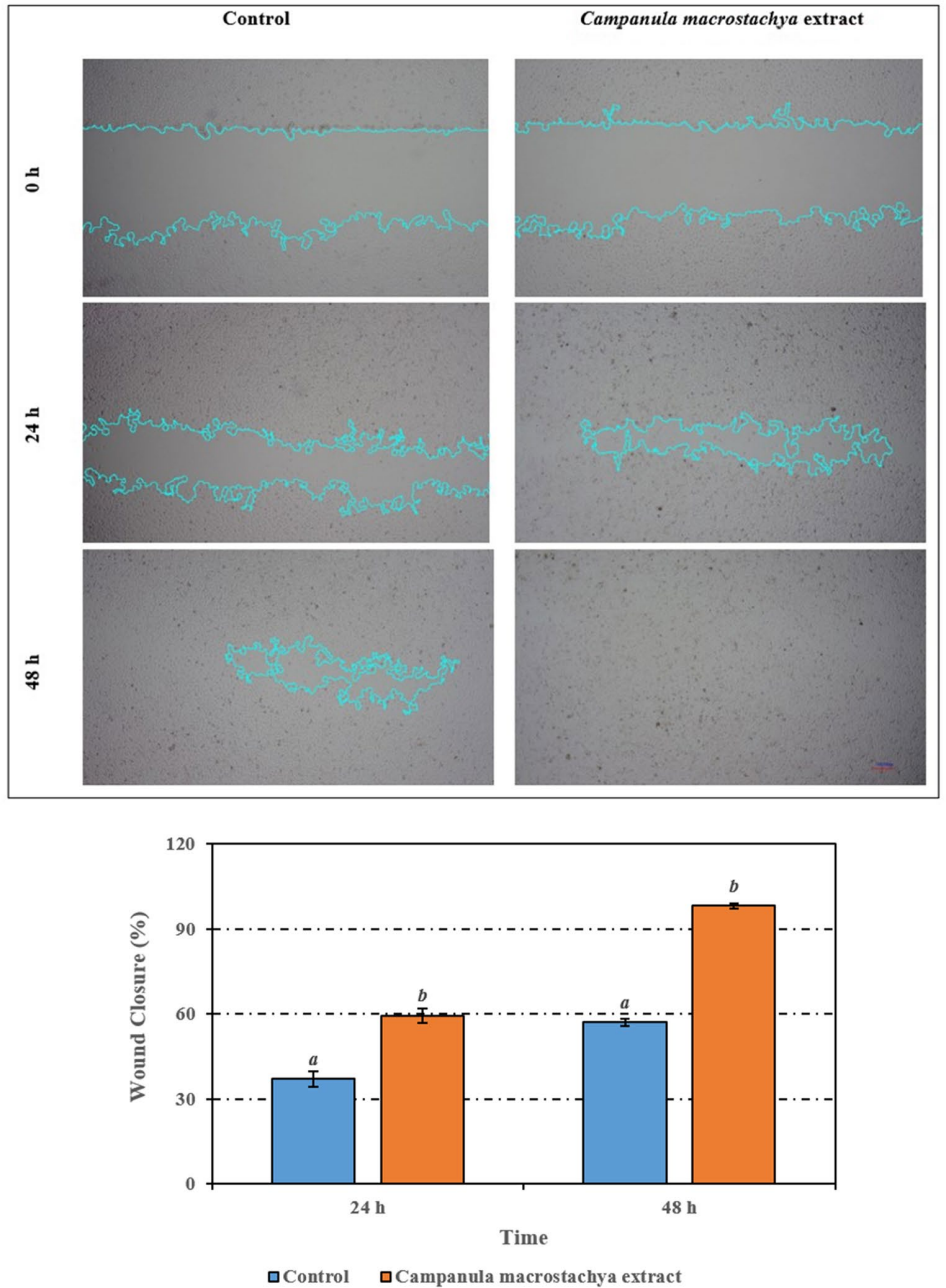
In vitro wound healing assay. Values are expressed as mean ± SD (*n* = 3), and mean values with different superscript letters indicate statistically significant differences compared to the control group, as determined by Student’s t-test (*p* < 0.05)

Figure 2 illustrates the representative images of the wound healing assay performed on HDFa cells at 0, 24, and 48 h. As shown, a progressive reduction in the scratch area was observed in both groups over time, indicating active cell migration and proliferation. However, the rate of wound closure was markedly enhanced in the extract-treated group compared to the control. After 24 h, the wound closure percentage was 37.00% ± 2.80 in the control group and 57.01% ± 1.42 in the extract-treated group. At 48 h, the wound closure rate in extract-treated HDFa cells (98.11% ± 0.90) was significantly higher than that of the control group (59.31% ± 2.52; *p* < 0.05).

### Cytokine modulatory effect

**Fig. 3:**
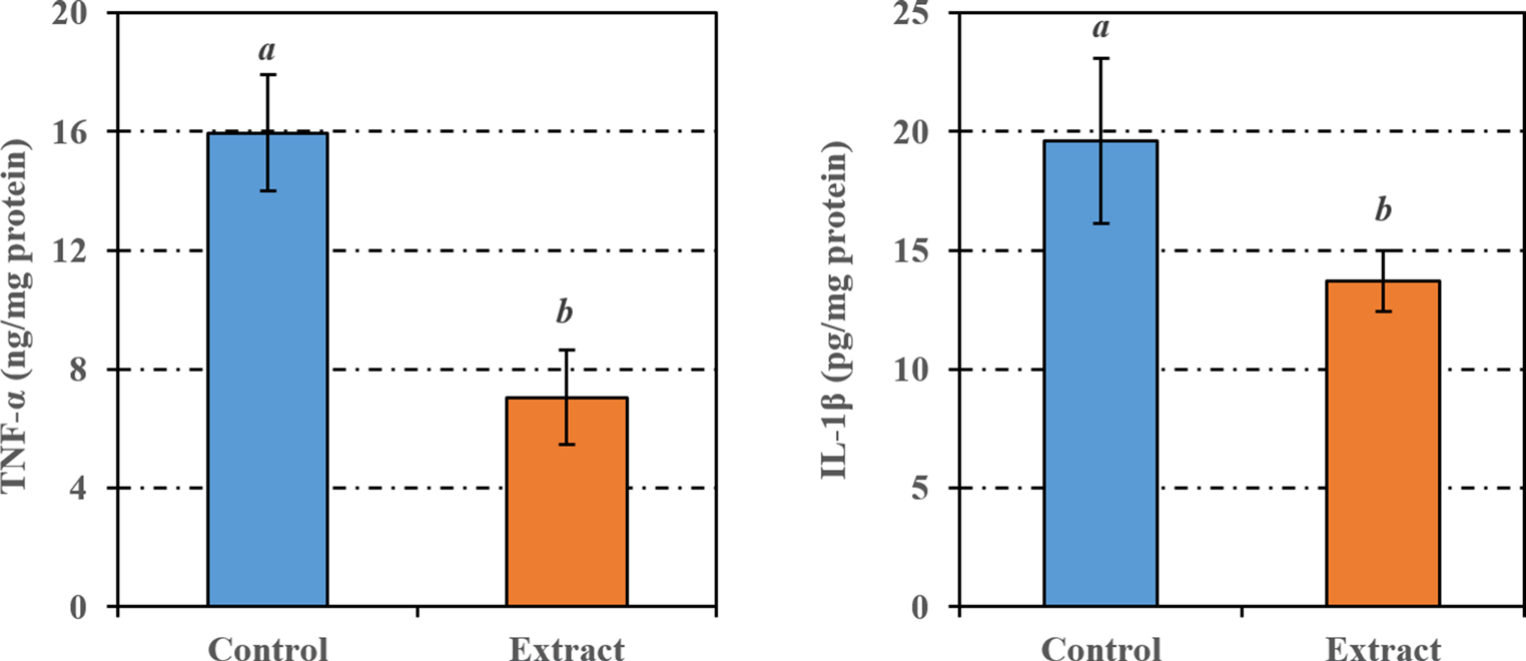
*Campanula macrostachya* extract on cytokine regulation. Values are expressed as mean ± SD (*n* = 3), and mean values with different superscript letters indicate statistically significant differences compared to the control group, as determined by Student’s t-test (*p* < 0.05)

Proinflammatory cytokine levels of TNF-α and IL-1β in HDF cells treated with *Campanula macrostachya* extract are shown in Fig. 3. While TNF-alpha was 7.06 ng/mL, IL-1 beta was determined to be 13.7 ng/mL. Results showed that levels of both cytokines decreased compared to the control group.

## Discussions

The statistical analysis of the *Campanula macrostachya* aqueous extract demonstrates a notable richness in phenolic acids, particularly chlorogenic and caffeic acids, which were identified as the dominant constituents. This phenolic dominance aligns with the findings of Sarikurkcu et al. (2021), where methanolic extracts of the same species contained comparable compounds—chlorogenic, hesperidin, and hyperoside—as the most abundant, collectively supporting the conclusion that *C. macrostachya* synthesizes a high proportion of hydroxycinnamic derivatives as its main antioxidant constituents. The total phenolic and flavonoid contents measured in the present study (46.76 mg GAE/g and 12.22 mg RE/g extract, respectively) were slightly higher than those previously reported for the methanol extract (22.83 mg GAE/g and 10.69 mg RE/g), which may be attributed to the enhanced extraction efficiency of the ultrasound-assisted aqueous technique that facilitates cell wall disruption and solvent penetration.

The present study provides a comprehensive assessment of the biological potential of *Campanula macrostachya*, situating its findings within the broader phytochemical and functional context of the *Campanula* genus. Consistent with previous reports, this species demonstrated strong antioxidant and selective enzyme inhibitory properties, confirming the pharmacological relevance of its phenolic constituents while also distinguishing it from closely related taxa in terms of activity profile and chemical composition.

In general, the antioxidant capacity observed for *C. macrostachya* aligns well with earlier data reported Sarikurkcu et al. (2021), who characterized the species as rich in phenolic acids—particularly chlorogenic and caffeic acids—responsible for its reducing and radical-scavenging effects. The current findings corroborate this, emphasizing that polar phenolic acids play a predominant role in the overall antioxidant response of the species. Moreover, compared with other *Campanula* taxa such as *C. baskilensis* (Yilmaz et al. 2023), *C*. *pelviformis* (Tsiftsoglou et al. 2023) and *C*. *macrostachya* (Sarikurkcu et al. 2021) displays a distinctive antioxidant pattern: rather than relying on diverse flavonoid derivatives, its activity appears to be primarily driven by hydroxycinnamic and hydroxybenzoic acid derivatives. This compositional difference explains why *C. macrostachya* shows stronger electron-transfer and radical-scavenging ability but relatively weaker metal-chelating efficiency than flavonoid-rich congeners.

When interpreted alongside the findings from *C. baskilensis* (Yilmaz et al. 2023) and *C*. *pelviformis* (Tsiftsoglou et al. 2023), a chemotaxonomic distinction becomes evident within the genus. While *C. baskilensis* exhibits broad-spectrum antioxidant activity attributed to hesperidin, nicotiflorin, and other flavonoid glycosides, *C. macrostachya* presents a more focused and potent activity derived from a smaller set of dominant phenolic acids. This difference may reflect adaptive chemical specialization linked to ecological or evolutionary factors, influencing both the secondary metabolism and functional bioactivity of these taxa.

The enzyme inhibition results further illustrate this chemical divergence. Although α-amylase and α-glucosidase inhibition does not constitute a direct wound-healing mechanism, these activities are biologically relevant in the context of delayed wound repair associated with metabolic dysregulation. Chronic hyperglycemia is known to impair wound healing by enhancing reactive oxygen species production, prolonging inflammatory cytokine release, and suppressing fibroblast migration and proliferation (Brem and Tomic-Canic 2007; Falanga 2005). By limiting postprandial glucose elevation, inhibition of carbohydrate-hydrolyzing enzymes contributes to the attenuation of glucose-induced oxidative stress and inflammatory burden, which are critical determinants of effective tissue repair (Apostolidis and Lee 2010; Tundis et al. 2010).

Phenolic-rich plant extracts that combine antioxidant capacity with α-glucosidase and α-amylase inhibitory activity have therefore been proposed as supportive agents in wound healing, particularly under conditions characterized by metabolic and inflammatory imbalance (Herman and Herman 2023; Sarikurkcu et al. 2020). In this respect, the selective α-glucosidase inhibitory activity observed for Campanula macrostachya may indirectly contribute to a cellular microenvironment that favors fibroblast function and wound closure, without implying a direct enzymatic role in the wound-healing process. In agreement with Sarikurkcu et al. (2021), *C*. *macrostachya* demonstrated meaningful inhibition of carbohydrate-hydrolyzing enzymes, particularly α-glucosidase, suggesting potential antidiabetic relevance through modulation of glucose absorption. Compared with *C. baskilensis* (Yilmaz et al. 2023), which shows multi-target enzyme inhibition due to its broader flavonoid profile, *C. macrostachya* exerts a more selective effect, reflecting the structure–function specificity of its phenolic acids. The predominance of chlorogenic and caffeic acids in the extract likely underpins this selective mechanism, as these compounds are known to interact with α-glucosidase active sites, mimicking carbohydrate substrates.

In contrast, the inhibition of cholinesterases and tyrosinase by *C. macrostachya* was less pronounced, supporting the conclusion that this species primarily acts on carbohydrate metabolism rather than neurological or pigment-related pathways. This trend is consistent with the observation that *C. baskilensis*—owing to its hesperidin and rutin content—exhibits stronger inhibition of cholinesterase and tyrosinase enzymes, indicating that the diversity and type of phenolic compounds largely determine the enzyme inhibition spectrum across *Campanula* species.

Collectively, these comparisons reveal that *C. macrostachya* represents a phenolic-acid–dominant model within the *Campanula* genus, exhibiting potent antioxidant and targeted α-glucosidase inhibition but limited activity against other enzyme systems. The data also reinforce that extraction methodology significantly influences bioactivity, as the aqueous ultrasound-assisted approach used here enhanced the recovery of polar phenolic acids without diminishing biological function.

The skin serves as a crucial protective barrier that shields underlying tissues from microbial invasion and environmental insults. When this barrier is disrupted, a rapid and coordinated wound healing response is essential to restore tissue integrity. Dermal fibroblasts play a pivotal role in this repair process by proliferating and migrating to the wound site. Cytokines such as TGF-β1 and TNF-α have been shown to promote fibroblast proliferation, keratinocyte migration, and re-epithelialization, thereby contributing to the overall wound healing process (Barrientos et al. 2008; Werner and Grose 2003). Furthermore, the pro-inflammatory cytokine IL-1β plays a crucial role during the early inflammatory phase by initiating the inflammatory cascade and regulating growth factor release, which in turn influences fibroblast activation and extracellular matrix remodeling (Eming et al. 2014; Strbo et al. 2013). Since fibroblast proliferation and migration are fundamental events in wound repair, exploring natural products and their bioactive compounds that modulate these cellular responses may provide valuable insights into improving cutaneous wound healing.

To ensure that the effects of the extract on fibroblast proliferation and migration were not influenced by cytotoxicity, cell viability was assessed after 24 h of treatment with various extract concentrations. The elevated MTT signal observed at 62.5 µg/mL should be interpreted as an increase in cellular metabolic activity, predominantly driven by dehydrogenase-dependent redox processes, rather than absolute cell proliferation, as phenolic compounds are known to enhance cellular redox metabolism without necessarily inducing cell division (Berridge et al. 2005; Riss et al. 2016). This concentration was therefore selected for subsequent wound healing assays.

The wound healing assay is a widely used and cost-effective method for screening natural products for their potential in vitro wound healing activity. This assay primarily reflects the proliferative phase of the wound healing process, which involves the migration and proliferation of keratinocytes and fibroblasts (Liu et al. 2000; Schäfer and Werner 2007). In the present study, an additional exogenous inflammatory stimulus was not applied to the cells; instead, the inflammatory response was evaluated within the in vitro wound healing model established using HDF cells. It is well documented that mechanical injury generated during the scratch assay induces a localized inflammatory microenvironment characterized by the release of pro-inflammatory cytokines and growth factors from fibroblasts. Accordingly, the modulation of the pro-inflammatory cytokines TNF-α and IL-1β following treatment with *C. macrostachya* extract reflects its potential regulatory effect on the inflammation phase of the wound healing process under in vitro conditions (Grada et al. 2017; Liang et al. 2007; Pastar et al. 2014). In parallel with these findings, the wound healing assay results clearly demonstrated that the plant extract significantly promoted fibroblast migration and proliferation, leading to an accelerated wound closure rate compared to the control group. The remarkable enhancement observed after 24 and 48 h suggests that the extract exerts bioactive effects that support tissue regeneration. Fibroblasts play a crucial role in wound repair by secreting extracellular matrix components and growth factors that facilitate re-epithelialization. Therefore, the enhanced wound closure response of HDFa cells in the presence of the extract indicates its potential to modulate cellular processes involved in wound healing, likely through a combined effect on cell migration and proliferation. These findings are consistent with previous reports demonstrating the wound healing potential of *C. macrostachya* extract, highlighting its ability to enhance wound closure in stimulated HDFa cells. These findings are consistent with previous reports demonstrating the biological activity of *Campanula* species. For instance, the methanolic extract of *C. lyrata* subsp. *lyrata* has been reported to significantly accelerate wound contraction and increase hydroxyproline content in excision and incision wound models, confirming its wound healing potential (Suntar et al. 2015). Moreover, *C. macrostachya* was recently characterized as a rich source of phenolic compounds exhibiting strong antioxidant activity, suggesting a possible contribution to its regenerative properties (Sarikurkcu et al. 2021).

Wound healing is a complex process consisting of four distinct phases: hemostasis, inflammation, proliferation, and remodeling. Cytokines are signaling molecules that mediate communication between immune cells and regulate fibroblast and epithelial cell activity in response to inflammatory stimuli (Moni et al. 2022; Wong et al. 2025). They are broadly categorized into pro-inflammatory and anti-inflammatory types. Pro-inflammatory cytokines such as TNF-α, IL-1, IL-6, and IL-8 stimulate immune activation, whereas anti-inflammatory cytokines such as IL-4, TGF-β, IL-1Ra, and IL-13 counteract inflammation by suppressing pro-inflammatory cytokine activity. Maintaining a balance between these cytokine groups is critical for proper wound repair (Wong et al. 2025).

Herbal products are known to possess diverse biological activities that can influence the biochemical mechanisms of wound healing (Herman and Herman 2023). For example, the extract of *Vitis vinifera* seeds has been shown to accelerate wound healing in rabbits by increasing TGF-β and VEGF levels while reducing TNF-α and IL-1β expression (Al-Warhi et al. 2022). Similarly, a cream containing *Aloe vera* and *Vitis vinifera* extracts exhibited significant wound healing activity by elevating VEGF and TGF-β1 in burn injuries (Moayeri et al. 2022). Silver nanoparticles incorporating *Resina draconis* and *Resina rosea* extracts reduced IL-1β, IL-6, TNF-α, iNOS, and MMP-9 expression while enhancing VEGF levels in diabetic mice (Ju et al. 2024). Extracts of Australian native plants (*Citrus aurantium*, *Mentha australis*, and *Eremophila longifolia*) have also been reported to reduce TNF-α and IL-6 levels in lipopolysaccharide-induced RAW 264.7 and HDF cells (Kapini et al. 2025). Likewise, the aqueous extract of *Roylea elegans* leaves decreased the pro-inflammatory cytokines TNF-α and IL-6 while increasing the anti-inflammatory cytokine IL-10 in Wistar albino rats (Upadhyay et al. 2021).

Consistent with these findings, our study demonstrated that the levels of the pro-inflammatory cytokines TNF-α and IL-1β decreased in the wound model established with HDF cells, suggesting that *C. macrostachya* extract may promote wound repair by modulating the inflammatory response.

However, the present study has several limitations. First, the biological evaluations were performed exclusively under in vitro conditions using human dermal fibroblast cells, and therefore the in vivo relevance of these findings remains to be clarified. Second, although the extract demonstrated significant effects on fibroblast viability, migration, and cytokine modulation, the molecular mechanisms and signaling pathways underlying these effects were not fully investigated. Third, the wound healing and cytokine assays were conducted using a single selected concentration, and dose-dependent mechanistic evaluations were not performed. Finally, the active constituents responsible for the observed biological effects were not isolated. Therefore, further studies involving bioassay-guided fractionation, molecular pathway analysis, and in vivo wound models are required to confirm the therapeutic potential and physiological relevance of Campanula macrostachya extract.

## Conclusions

This study provides the first comprehensive evaluation of the phytochemical composition and in vitro wound-healing potential of the aqueous extract of *C. macrostachya* obtained via ultrasound-assisted extraction. The extract was found to be rich in phenolic acids, particularly chlorogenic and caffeic acids, which are likely to contribute to its antioxidant and enzyme inhibitory properties.

Under in vitro conditions, the extract enhanced fibroblast metabolic activity and significantly accelerated wound closure in human dermal fibroblast cells. In addition, the observed reduction in pro-inflammatory cytokines TNF-α and IL-1β suggests that the extract may contribute to the regulation of inflammatory responses associated with the wound healing process. These findings indicate that the biological activity of C. *macrostachya* extract may be associated with its phenolic composition and its capacity to modulate cellular redox balance and inflammatory signaling.

Overall, the results support the ethnopharmacological relevance of *C. macrostachya* and suggest that its aqueous extract may represent a promising source of bioactive compounds for further investigation in skin-related applications. However, additional mechanistic studies and in vivo investigations are required to confirm its therapeutic potential and to clarify its physiological relevance.

## Supplementary Information

Below is the link to the electronic supplementary material.

Supplementary Material 1

## Data Availability

Raw data is available upon request by reviewers.
